# Complete Genome Sequence of an Enterobacter roggenkampii Strain with Reduced Carbapenem Susceptibility Isolated from a Home-Visit Nursing Agency

**DOI:** 10.1128/mra.00353-22

**Published:** 2022-08-16

**Authors:** Yusuke Ota, Chihiro Tani Sassa, Masayo Kashiwagi, Chikako Okawara, Shuji Tohda, Ryoichi Saito

**Affiliations:** a Department of Molecular Microbiology, Graduate School of Medicine and Dental Science, Tokyo Medical and Dental University, Tokyo, Japan; b Department of Clinical Laboratory, Tokyo Medical and Dental University Hospital, Tokyo, Japan; c Department of Infection Control and Prevention, Tokyo Medical and Dental University Hospital, Tokyo, Japan; d Department of Innovation in Fundamental and Scientific Nursing Care, Graduate School of Health Care Sciences, Tokyo Medical and Dental University, Tokyo, Japan; e Department of Laboratory Medicine, Tokyo Medical and Dental University, Tokyo, Japan; Loyola University Chicago

## Abstract

Carbapenem-resistant bacteria represent an emerging threat to global health; nursing homes may be reservoirs for these isolates, which cause life-threatening infections. Here, we present the complete genome sequence of an Enterobacter roggenkampii strain with reduced carbapenem susceptibility that was isolated from a sink in a home-visit nursing agency.

## ANNOUNCEMENT

The emergence of carbapenem resistance has become a public concern. The environment plays a key role in the dissemination of carbapenem-resistant bacteria, including Enterobacter spp. (1). Nursing homes are unique environments associated with challenges for infection control because antibiotic-resistant isolates are endemic in such facilities and hospitals (2–4). The genomic characterization of carbapenem-resistant strains from such facilities is necessary for a better understanding of the molecular epidemiology and genetic basis and for preventing dissemination.

Here, we detected an Enterobacter cloacae complex strain, which was identified initially by matrix-assisted laser desorption ionization–time of flight mass spectrometry (Bruker Daltonics GmbH, Bremen, Germany), after aerobic incubation overnight at 35°C on CHROMagar mSuperCARBA agar (Kanto Chemical, Tokyo, Japan), isolated from a sink surface swab in a home-visit nursing agency in Tokyo, Japan. The MIC of imipenem was 2 μg/mL, indicating intermediate antibiotic resistance (5). Total DNA was extracted using a NucleoBond high-molecular-weight (HMW) DNA kit (Macherey-Nagel, Düren, Germany) per the manufacturer’s instructions. Illumina 2 × 256-bp paired-end sequencing reads were generated using a Nextera DNA flex library prep kit (Illumina Inc., San Diego, CA) and the Illumina MiSeq platform. Genomic libraries for Oxford Nanopore long reads were generated using a short read eliminator XS kit (Circulomics Inc., Baltimore, MD) and ligation sequencing kit (Oxford Nanopore Technologies, Oxford, UK); sequencing was performed using the R9.4.1 flow cell and Nanopore GridION sequencer (Oxford Nanopore Technologies). Raw short and long reads were quality filtered using Fastp v0.20.1 with parameters “-q 30 -n 20 -t 1” (6) and NanoFilt v2.7.1 with parameters “-q 10 –headcrop 50 -l 1000” (7), respectively. *De novo* hybrid assembly of the filtered reads was performed using Unicycler v0.4.8 (8). The genome was annotated using NCBI PGAP v6.1 (9). Bacterial species, sequence type (ST), and antibiotic resistance genes were identified using BLAST (10), MLST v2.0 (11), and CARD (12), respectively. Unless otherwise noted, default parameters were used for all software tools.

A total of 2,926,466 short reads and 177,665 long reads (*N*_50_ size of 20.15 kb) with an estimated genome coverage of 448.7× were sequenced, and the complete genome was constructed as a chromosome containing 4,740,531 bp with a GC content of 55.7% and 11 plasmids ranging in size from 3,360 bp to 115,683 bp. The chromosome sequence was best matched to that of Enterobacter roggenkampii DSM 16690 (GenBank accession number CP017184), with an average nucleotide identity value of 98.5%; the isolate was classified as ST486. Resistome analysis showed that the chromosome had an MIR-22-type β-lactamase and AcrR multidrug efflux pump genes (with 100% identity in the CARD database), which may contribute to imipenem resistance (13). The MIR-type *E. roggenkampii* with reduced carbapenem susceptibility has been isolated from blood cultures in Japan; thus, our isolate may have spread from the hospital via environmental sources or the medical staff (14). Our data provide a basis for understanding the appearance of bacteria with reduced carbapenem susceptibility in nursing homes; thus, serial monitoring of environmental contaminations of the isolate is necessary to control infections caused by these bacteria, especially in clinical settings.

### Data availability.

These genome data of the *E. roggenkampii* strain have been deposited in GenBank under the accession number SAMN27117675. The NCBI assembly number is GCA_023195715.1. Raw data are available in the SRA accession numbers SRX14775294 and SRX14775295.
